# Male Involvement in Reproductive and Maternal and New Child Health: An Evaluative Qualitative Study on Facilitators and Barriers From Rural Kenya

**DOI:** 10.3389/fpubh.2021.644293

**Published:** 2021-04-21

**Authors:** Adelaide M. Lusambili, Peter Muriuki, Stefania Wisofschi, Constance S. Shumba, Michaela Mantel, Jerim Obure, Lucy Nyaga, Kennedy Mulama, Anthony Ngugi, James Orwa, Stanley Luchters, Marleen Temmerman

**Affiliations:** ^1^Department of Population Health (DPH), Aga Khan University, Nairobi, Kenya; ^2^Centre of Excellence in Women and Child Health, Aga Khan University, Nairobi, Kenya; ^3^Department of Public Health and Primary Care, International Centre for Reproductive Health, Ghent University, Ghent, Belgium; ^4^Department of Epidemiology and Preventive Medicine, Monash University, Melbourne, VIC, Australia; ^5^Department of Obstetrics and Gynaecology, Medical College, Aga Khan University, Nairobi, Kenya

**Keywords:** gender, barriers, facilitators, RMNCH, Kenya

## Abstract

Male involvement in reproductive, maternal, newborn and child health (RMNCH) is known to improve maternal and child health outcomes. However, there is sub-optimal adoption of male involvement strategies in several low- and middle-income countries such as Kenya. Aga Khan University implemented Access to Quality of Care through Extending and Strengthening Health Systems (AQCESS), a project funded by the Government of Canada and Aga Khan Foundation Canada (AKFC), between 2016 and 2020 in rural Kisii and Kilifi counties, Kenya. A central element in the interventions was increasing male engagement in RMNCH. Between January and March 2020, we conducted an endline qualitative study to examine the perspectives of different community stakeholders, who were aware of the AQCESS project, on the facilitators and barriers to male involvement in RMNCH. We found that targeted information sessions for men on RMNCH are a major facilitator to effective male engagement, particularly when delivered by male authority figures such as church leaders, male champions and teachers. Sub-optimal male engagement arises from tensions men face in directly contributing to the household economy and participating in RMNCH activities. Social-cultural factors such as the feminization of RMNCH and the associated stigma that non-conforming men experience also discourage male engagement.

## Introduction

The 1994 International Conference on Population and Development in Cairo and the 1995 World Conference on Women in Beijing marked the significant recognition that women's empowerment requires the engagement of men to advance positive improvements in reproductive, maternal, newborn and child health (RMNCH) outcomes. The Beijing Declaration noted a broad consensus to “encourage men to participate fully in all actions toward equality” (1). This intersectional approach demands meaningful male involvement to uncover and address male attitudes, beliefs and practices that sustain gendered power differentials and reinforce inequalities between women and men.

Men have the potential to act as allies, partners, and co-beneficiaries in women's empowerment by recognizing that restrictive gender norms affect both women and men (1). Conventional gender norms that label men with good health service seeking behavior as “weak” may discourage healthy and positive behaviors and ultimately affect men's health-related outcomes (2–4). Further, men play a critical role in the overall health of the family as men's understanding of reproductive, maternal, new-born and child health (MNCH) affects women's access to and uptake of RMNCH health services. In many low- and middle-income settings men determine women and children's access to and utilization of health services with significant implications on their health outcomes (5–8).

In rural Kenya, women traditionally live in social, cultural, and economic structures that espouse patriarchal ideologies and women's disempowerment through limited control over the socio-economic and health features of their lives. This means that men must be encouraged to become active and educated consumers of health services, even those that may not traditionally be of male interest such as RMNCH services (1, 2). Participants from Tanzania and Zimbabwe reported that both men and women valued men's involvement in RMNCH, especially pertaining to positive improvements in maternal outcomes, reduced maternal workload, and increased couple communication (6–8).

Previous research has also demonstrated that in the absence of partner communication, women may incorrectly assume that their partners or spouses oppose family planning and/or RMNCH services (4). Poor communication between husbands and wives, spouses' lack of knowledge of RMNCH topics, social stigma, and competing priorities have all been cited as barriers to male involvement in RMNCH (9, 10). However, a program in India found that targeted outreach and sensitization of men through the integration of male community health volunteers (CHVs) reinforced and complemented the work of female CHVs leading to increased demand for RMNCH services in the communities (11). Given that men's understanding of services may both directly and indirectly affect women's utilization of services, both women and men should be fully and actively engaged in RMNCH interventions.

While benefits of male engagement have been documented and several programs have been implemented in diverse settings to promote male engagement, there remains a need to understand facilitators and enablers of male engagement in RMNCH in the rural Kenyan context. Our research study investigated the facilitators and barriers to working with men to promote RMNCH in partnership with women. We provide practical programmatic recommendations through a critical reflection of gender and relationship dynamics and how men's attitudes surrounding male involvement in RMNCH interventions may be leveraged to strengthen program objectives.

## Methods

### Study Design

A qualitative study.

### Study Setting

The evaluation study was conducted in AQCESS implementation target areas of Kilifi and Kisii counties. Detailed social–cultural characteristics of the study area can be found in our previous papers (12, 13).

### Methods

We conducted 10 focus group discussions (FGDs) and 11 key informant interviews (KIIs) across the two study sites. All participants were individuals who were familiar of the AQCESS project and had lived in the AQCESS area in the past 1 year. Participants were purposively sampled by AQCESS field project coordinators. Key informants were males and females at the county, sub-county and health facility levels. FGDs were conducted separately with female and male CHC members, male and female adult community members, and female and male adolescent community members. The qualitative evaluation explored their perceived and observed barriers to male participation in RMNCH services at the household and at the community levels as well as factors that may hinder men participation.

### Interview Process

Data collection was led by a Kenyan qualified social scientist with over 15 years of experience in research, a qualified qualitative consultant and a team of experienced research assistants. The study was conducted between January and March 2020 after ethics clearance from the Aga Khan University (AKU) and the National Commission for Science Technology and Innovation (NACOSTI/P/19/2768). Additional permission and consent for this study was sought from the local communities including the local Health Facility (HF) and CHCs in both the study sites.

Before commencing data collection, research assistants were trained on the approved protocol requirements. Parental/guardian assent was sought for adolescents <18 years of age. All participants provided written consent prior to participating in the study. Interviews were collected in local Swahili language, face to face and after providing the study participants with full disclosure and information on the study objectives, risks and benefits. Interviews and FGDs were conducted with facilitators who were the same gender. Interviews were conducted in the community spaces including facilities and schools that were deemed convenient and confidential for interviews to share their experiences.

### Data Management and Analysis

All interview audio recordings and reflective notes were transferred to a password protected computer at AKU's Monitoring Evaluation and Learning Unit (MERL). Audio recordings were deleted after all data was transcribed verbatim by a qualified transcriber from Swahili to English. During transcription process, all transcripts were anonymized by deleting all identifiers such as names to safeguard participants' confidentiality. Translated and transcribed data was checked by the study PI and study consultant who are Swahili native speakers. All the transcripts and reflective notes were uploaded into NVivo 12 Data Analysis Software.

To address reliability and validity, first, the research team, conversant in both Kiswahili and English randomly selected a few audio recordings and complimenting transcripts to verify as well as validate the content of the transcribed data. Secondly, a quarter of the transcripts was coded by two researchers after which they developed a coding framework that was used to code the remaining data, identify categories and main themes.

A data interpretation workshop was held with the research team and the key stakeholders in the AQCESS implementing areas and some of the barriers and facilitators to male engagement presented in this paper were confirmed during these sessions as illustrated in the following table.

## Codes, Categories, and Themes

**Table:** 

	**Codes**	**Categories**	**Themes**
*A*	1. Alcoholic men; 2. Early morning sessions suitable for alcoholic men; 3. RMCH sessions at church; 4. Regular visits by CHVs 5. Male listen to male leaders; 6. Male authorities' messages respected; 7. Male authority messages impactful; 8. Provide stipends for men.	1. Morning sessions for male; 2. CHVs role; 3. Use of male leaders; 4. The role of the church; 5. Stipend and /incentives.	**Facilitators** 1. Time sensitive information Sharing for men 2. The role of male leaders /influencers 3. The role of the CHVs and church sessions 4.Incentives
*B*	1. Men are breadwinners; 2.Men start work in the morning; 3. RMCH sessions conflicts with men's economic activities; 4. Men have no time; 5.RMNCH is a woman's affair; 6. Men devalued for participating in RMNCH activities; 7.Men are stigmatized; 8. Men lack knowledge.	1. Mens economic roles conflicts with RMCH activities; 2. Stigma and social cultural perceptions 3. Males lack of knowledge.	**Barriers** 1. Economic Barriers 2. Feminization of RMNCH –linked to social cultural perceptions and Men's lack of knowledge.

## Findings

Key enablers to male engagement included individual and couple information sharing, and the use of community male influencers, while barriers were socio-cultural and economic factors, peer pressure, drunkenness and men's low educational attainment.

### Facilitators to Effective Male Engagement

Participants were asked to share their opinion on factors that encouraged male involvement in RMNCH. They mentioned among others; the importance of information sharing on the community health status with men, as to engage them in being part of the solution, and the importance of targeting male education sessions at home early in the morning, as this is typically when men are at home prior to taking on tasks outside the homestead.

*I can say this, mostly, those [men] who are not alcoholics or have been, these teachers go to teach these people before they take alcohol and they get to listen to them… Yes, at home before going to take alcohol. The man knows that there is something good here. And those who attend churches are being taught from there on the importance of having a family full of love. If the mother falls sick, the husband carries the burden. So, the church teaches that. And also, those teachers [CHVs] who teaches us at home, they should come before one goes to take alcohol. So even if he doesn't understand on the spot, they [CHVs] should continue visiting him, or during the baraza* [community dialogue session], *he gets that information there. Just like that*.

***FGD_Male community members, Kisii***

Most importantly, the participants highlighted that men were more receptive to messages that come from influential male leaders in the community such as church leaders, male champions and teachers. The advice from these authority figures was reported as being impactful in promoting male involvement.

*They were supportive and you know men, men listen more to, to the authorities. So, when something is suggested in chief baraza [community gathering] it becomes owned by men. Men own it more….and training of male champions, where you appreciate that their men who actively take part in RMNCH services or promotion*.

***KII_County gender representative, Kilifi***

Sharing from their observed experience, participants in both Kisii and Kilifi explained that offering men a small stipend after attending community meetings may motivate them to participate in RMNCH initiatives.

*Like when they attend the forums, then after the forums, they may be given incentives to give them morale*.

***FGD_Community Health Committee for women, Kilifi***

*Any other factors that make it easier, is about, you know men they love that stipend that they usually being given… Yes, at the end of each committee there is a small stipend that they are being given so it motivates them a lot, but it cuts across both men and women*.

***KII_Health Facility manager, Kisii***

In both Kilifi and Kisii, transportation costs may be a barrier to attend RMNCH health activities, which is compounded with the challenge of balancing informal jobs and prior commitments with attending gender sensitive community seminars. This may imply that men, who are the financial providers for their families, have to choose between losing their income and attending sessions. Stipend may be one method of encouraging them to participate in community meetings as it may be used for transport costs as well as be allocated toward the purchase of household's essentials. However, stipends such as daily allowance for seminar attendance, transport reimbursements and meal vouchers are not sustainable in many instances after the donors have concluded the projects.

In Kisii, specific days (christened “couples day”) set aside at health facilities to counsel couples on health matters were referenced as a facilitator of male involvement, as they increased men's knowledge of RMNCH. These couples' days were hosted at the health facilities, where couples were invited to jointly attend and received information directly from health care workers on RMNCH. As a result, some men became RMNCH champions, demonstrating that this targeted intervention was effective at increasing men's knowledge and support for RMNCH.

*Couple's day. On this event they inform us on what is supposed to be done. Even though it is difficult, that brings about a change. Let's say if he went with his wife, and you didn't attend, he will tell you the importance and how he became the champion of the day because we went with my wife and we were told this and that. And it is very easy. In fact, they have tried to enlighten men during this couple's day and they are able to see good progress*.

***FGD_Male community members, Kisii***

### Barriers to Effective Male Engagement

Respondents were asked to reflect and share their views on barriers to effective male involvement. Economic and social cultural barriers were referenced and discussed.

**Economic barriers**, such as men prioritizing taking part in casual jobs over attending community activities, reflect the tensions that men face in balancing their roles as providers and trying to keep abreast with participating actively in RMNCH activities, and related roles and decisions at the community and household levels. Often the need to take on casual jobs to financially contribute to their families was an overriding priority over attending these scheduled RMNCH activities.

*Then the other aspect, it is economical aspect, where men are the bread winners in the family, most families, so, getting men taking part in activities sometimes became a big issue because of lack availability of the same, come for maybe meetings or even accompanying their wives and this kind of things, yes*.

***KII_County gender representative, Kilifi***

*It may happen that you have nothing in the pocket needed at home to feed the family and then you are called for a casual job. If like the dialogue is to begin like at 10 and you are called for a casual job, that may take hours or maybe 4 days, so it's challenging, you will decide to go to that job at least get your family something to feed and when I get a chance I will go the next meeting, so, that brings a challenge*.

***FGD_Male community members Kilifi***

*They would say they are busy, they have gone to search [look for jobs] for the family and then men value their time, men value their time so that they are called at eight* *(**8**)* *and the engagement is at eleven* *(**11**)* *many of them would have already gone away*.

***KII_County gender representative, Kisii***

As these narratives demonstrate, the low socio-economic status of communities in Kilifi and Kisiii is an important consideration, demonstrating that male engagement in RMNCH activities must be accompanied with sustainable economic activities embedded in the social cultural context which will reduce the tensions men face in simultaneously trying to attend RMNCH seminars and contribute to the household economy. Some male CHVs reported feeling reluctant to participate in RMNCH activities as there was little to no buy–in for their time contribution. So long as there is no pathway for sustainable economic support, for example the consistent provision of stipends, sustainable male engagement is threatened.

**Social-cultural factors such as the feminization of RMNCH issues** were reported as barriers to male engagement. Men participating in RMNCH feared being viewed as “weak” by their peers. This discouraged some men from participating in what was perceived to be women's issues. Further, there was significant peer pressure and stigma associated with non-conformity that led men to shy away from being involved in RMNCH activities. Men who take on “care work” or “women's work” are perceived to be weak. For instance, it would be considered unacceptable for a man to carry a child as this is viewed as a woman's role. In this situation, the man would be perceived as the weaker partner in the relationship. This situation illustrates the underlying deep-seated gender inequitable attitudes stemming from the social and cultural norms that impede men's participation in RMNCH. These barriers are captured in the following quotes from Kisii:

*Most of them have stigma. Another contributing factor is peer group pressure and cultures. These are the most affecting things in dragging the men behind instead of moving forward in helping the women and taking care of the women during the pregnancy period…. Tradition like we us Kisii we have our own tradition and we have others who also have their own tradition. For the Kisii they say that is the responsibility of the woman let her go to the hospital after she is done, she will come back home. Men do go out to do their own things*.

***FGD_CHC men, Kisii***

*Attitude, attitude…They know matters of health care is to the woman and the children, is to the woman and the baby*.

***KII_County gender representative, Kisii***

*Yes, another one is culture, just as I explained earlier like a man, you are not supposed to be seen going to the hospital every now and then, for that you will be considered weak, yes, so culture also contributed*.

***KII_County gender representative, Kisii***

Similar sentiments were also reported from Kilifi.

The barriers are social cultural issues like men being that the issue of RMCAH services is predominantly a women's' issue…

***KII_County gender representative, Kilifi***

…*why he would be going to the clinic regularly, as if, have you become a woman? Such will easily demoralize you…, …if we would listen and follow on what we hear from other people [men], they we wouldn't have accompanied them…because we are told so many things, you have been overruled, you carrying a baby and yet you have a wife, are you the one who married or you got married by her?*

***FGD_Male community members Kilifi***

By and large, in Kisii, social cultural factors intersected with inadequate knowledge, feigned unavailability and ignorance, which made it difficult for women to be supported by their partners/spouses in attending to household chores and RMNCH services, as the below quotes demonstrate.

*Yes, I think its ignorance. Male parents are ignorant, even if you advise them, they are also supposed to do family planning or take their wives to clinics for family planning, they just ignore. Because of that ignorance they just look at it as a woman responsibility. It's partly, there is ignorance, and a man would ignore and say that that is women's duties, for those who have not been enlightened*.

***FGD_CHC women, Kisii***

…* Inadequate knowledge I can call it, because majority of the male person there for one reason or the other dropped out of school. And many of those who dropped out of school now are the motor rider, motorcycle riders or some like Bomachoge Borabu borders the Masaai, many of them go to the Masaai place in terms of pretense of farming, so they are normally not very available*.

***KII_County gender representative, Kisii***

*There are men who say, in our culture, men are not supposed; so that has made it difficult. Two, is the peer group. The bad company where you are with other men who tells you that if your woman tells you to help her do something then she must have bewitched you. Three, ignorance. We know but we don't want to do. Then lastly, even though it is in the category of peer group, is drunkenness. Alcohol makes this work to be difficult*.

***FGD_Male community members, Kisii***

## Discussions

This study examined the perspectives of various stakeholders on the facilitators and barriers to male engagement in RMNCH and not only increased our knowledge and understanding of these, but also exposed potential ways of addressing the existing gaps. Key facilitators to male engagement include individual and couple information sharing. We also identified socio-cultural and economic factors, peer pressure, drunkenness and the low educational background of men as barriers to effective male engagement.

Directly sharing information on the health status in the community with men and doing so at convenient times for men, for instance, before they leave their homes for work is an enabler for effective male engagement. This is consistent with other studies that have shown that providing men with information and involving them in counseling sessions improves support and decision-making for RMNCH (11). In addition, couples' day, an innovative gender-transformative approach involving joint counseling of couples on RMNCH run at the health facilities, was a major facilitator to male engagement.

We identified targeted information sessions for men on RMNCH as a key facilitator of effective male engagement. Further, information delivery was even more impactful when conveyed by male authority figures in the community such as church leaders, male champions and teachers. An innovative approach using the couples' day run at the health facilities, where couples were invited to jointly attend and directly receive information from health care workers on RMNCH was also effective at increasing men's knowledge and support for RMNCH.

A key barrier to effective male engagement was the tension between men's financial responsibilities and their role in contributing to the household economy and the need to participate in RMNCH activities and decision-making. Competing priorities hindered their ability to access information on RMNCH, participate in related activities and make informed decisions. This is consistent with findings from Kenya, where the majority of young women aged 15–24 from several counties reported that their husbands were occupied with work responsibilities and lacked the time to participate in RMNCH activities (14). Thus, it is important to highlight the link between RMNCH and the household economy by providing evidence that the household's financial situation could also be improved as a result of the increased well-being of women and children. An additional implication of this was the importance of intervention timing and scheduling. We found that it was preferred to conduct the health education prior to men leaving their homes early in the morning.

Further, drunkenness was also referenced as a barrier to male engagement as it created additional challenges to reach men with information, effectively engage them on RMNCH and increase their self-efficacy to implement the recommended actions to derive the maternal and child health benefits. This continues to highlight the importance of engaging men at the right time, when they are sober, to achieve the required support and attitudinal changes. There is also need to implement supportive programs, such as alcohol abuse prevention and control interventions, in the communities alongside RMNCH programs to ensure the effectiveness of the proposed RMNCH programs and the creation of enabling family and community environments.

Social-cultural factors such as stereotyping and the feminization of reproductive and maternal health issues presented an additional barrier threatening effective male involvement. Such factors coupled with stigma and peer pressure may hinder even the most well-meaning educational programs targeting men. This finding aligns with others who have found that conventional gender norms that label men's positive health service seeking behavior as “weak” may discourage adoption of healthy and positive behaviors (2–4, 14). To overcome this, broader gender transformative programs are needed at the community level that integrate increased sensitization and promote gender equality alongside male involvement efforts. Community-based awareness programs may normalize this behavior as a way of transforming negative attitudes by depicting men who care for their families through active involvement in RMNCH as “strong” men who love and protect their families. An example that proved to be successful in this study setting was the couples' day approach run at the health facilities that involved joint counseling of couples on RMNCH.

In addition to the socio-cultural factors, ignorance attributed to the low educational status of men was also reported as a barrier to male engagement. This concurs with findings in Nepal where men's educational background was linked to their degree of engagement in RMNCH (15). In this study, men with higher literacy levels were more likely to make informed choices regarding reproductive health than those with lower educational levels. One implication is the important of promoting higher educational attainment for men living in low resource settings and integrating gender equity topics in school curriculum to foster positive attitudes and encourage gender equality from a younger age. Secondly, effective demand-side behavior change and communication strategies must be tailored to men with lower educational levels to improve their ability to participate in meaningful ways in RMNCH programs and address the persistent gender inequities that perpetuate poor RMNCH outcomes.

Policy implementation could include salaried male community health volunteers, income generating activities for sustainability purposes, and introducing a RMNCH syllabus that also promotes the importance of male engagements in RMNCH activities to adolescents in the local schools. Our findings reinforce the importance of male involvement in RMNCH programs and the potential this gender transformative approach has in positively influencing maternal and child health. The study supports similar findings that highlight the positive influence of gender transformative approaches on RMNCH outcomes (5).

The strength of this study is that we interviewed stakeholders who were directly linked to the project activities. This allowed them to provide narratives grounded in their lived experiences during project implementation. The voice of individuals who were direct beneficiaries of project activities is powerful in policy implementation. The limitation of this study is that the study team only focused on the AQCESS implementation areas which may inflict biases to our findings. Future evaluative studies should consider including non-beneficiary, control, areas to compare the efficacy of the findings.

## Conclusions

The findings underscore the critical importance of male involvement in RMNCH programs as well as the need to continually address the underlying gendered attitudes that hinder men's participation in RMNCH. It is important to strengthen and sustain targeted male involvement and education programs to increase men's knowledge and support for RMNCH. Working alongside key male authority figures in the community may have the greatest impact in increasing men's involvement. This study has contributed to defining and unpacking the barriers, facilitators and benefits of male involvement in RMNCH programs in rural Kenya, and provides evidence for building and improving similar gender transformative approaches in RMNCH projects. Other studies are required to assess the feasibility and acceptability of adaptations of male involvement programs that are co-designed with the communities.

## Data Availability Statement

The datasets presented in this article are not readily available because there may be restrictions to the data as some of the narratives have identifying variables. However, researchers who meet the criteria for access to confidential data can contact the main authors. Requests to access the datasets should be directed to Adelaide M. Lusambili, adelaide.lusambili@aku.edu.

## Ethics Statement

The studies involving human participants were reviewed and approved by both the Institutional Review Board of Aga Khan University, Kenya and National Commission for Science, Technology, and Innovation (NACOSTI) (NACOSTI/P/19/2768) on 03/December 2019. Written informed consent to participate in this study was provided by the participants' legal guardian/next of kin.

## Author Contributions

AL: conceptualization, data collection, data analysis, supervision, writing, visualization, and validation. PM: data collection, data analysis, data interpretation, writing, and review of the final draft. SW: formal analysis, interpretation, writing, visualization, and validation. CS: data interpretation, writing, visualization, and validation. MM: acquisition, project implementation and management, data interpretation, and manuscript review. JO: conceptualization of research, data interpretation, manuscript review, and validation. LN and KM: coordination and infrastructural support for actual study implementation. SL: manuscript review, visualization, and validation. AN: conceptualization and development of study tools. MT: overall PI of the AQCESS implementation and the MERL Unit. All authors contributed to the article and approved the submitted version.

## Conflict of Interest

The authors declare that the research was conducted in the absence of any commercial or financial relationships that could be construed as a potential conflict of interest.

## Abbreviations

AKFC: Aga Khan Foundation Canada
AKU: Aga Khan University
ANC: Antenatal care
AQCESS: Access to Quality Care through Extending and Strengthening Health Systems
CHC: Community Health Committee
CHU: Community Health Unit
CHV: Community Health Volunteer
FGD: Focus Group Discussion
FP: Family Planning
HF: Health Facility
HIC: High-Income Countries
KII: Key Informant Interview
LMIC: Low- to Middle-Income Countries
MERL: Monitoring and Evaluation and Research Learning
MNCH: Maternal, Newborn & Child Health
NACOSTI: National Commission for Science, Technology and Innovation
PI: Principal Investigator
PNC: Post-natal care
RMNCH: Reproductive, Maternal, Newborn, Child and Adolescent Health
SSA: sub-Saharan Africa.
